# Role of Ultrasonography and Magnetic Resonance Imaging in Diagnosing Shoulder Pathologies: A Prospective Observational Study

**DOI:** 10.7759/cureus.106977

**Published:** 2026-04-13

**Authors:** Rohini Chaudhari, Pranit B Pantawane, Sourabh Zambre, Pankaj Badarkhe, Payal M Badarkhe

**Affiliations:** 1 Radiology, Indira Gandhi Government Medical College, Nagpur, IND; 2 Radiology, NKP Salve Institute of Medical Sciences and Lata Mangeshkar Hospital, Nagpur, IND; 3 Neurosurgery, All India Institute of Medical Sciences, Nagpur, IND; 4 Radiology, Government Medical College, Akola, IND; 5 Biochemistry, Dr Panjabrao Alias Bhausaheb Deshmukh Memorial Medical College, Amravati, IND

**Keywords:** labral lesions, magnetic resonance imaging, rotator cuff tear, shoulder pain, ultrasonography

## Abstract

Background

Shoulder pain is a common musculoskeletal complaint with diverse etiologies involving rotator cuff, labral, and periarticular structures. Accurate imaging is essential for diagnosis and management. Ultrasonography (USG) and magnetic resonance imaging (MRI) are widely used modalities, each with distinct advantages and limitations. The aim of this study was to compare the diagnostic performance of USG and MRI in evaluating shoulder pathologies, particularly rotator cuff tears, using surgical or arthroscopic findings as the reference standard where available, and to assess their complementary roles in clinical practice.

Methods

This prospective observational study included 75 patients presenting with shoulder pain, restriction of movement, or instability. All patients underwent USG and MRI of the affected shoulder using standardized protocols. USG and MRI examinations were interpreted independently by experienced musculoskeletal radiologists. Readers were blinded to the findings of the other imaging modality at the time of reporting; however, relevant clinical history regarding the symptomatic side was available, consistent with routine diagnostic practice. Radiologists were not blinded to surgical outcomes at the time of final correlation, as surgical findings were used as the reference standard in the operative subgroup. Imaging findings were compared between modalities and correlated with surgical or arthroscopic findings where available. Diagnostic performance parameters, including sensitivity and specificity, were calculated in the surgically verified subgroup (n=34). Interobserver agreement for USG and MRI interpretations was not formally assessed. Statistical analysis was performed using the chi-square test, with p<0.05 considered statistically significant.

Results

The study population showed a male predominance (52/75; 69.3%) and greater involvement of the right shoulder (54/75; 72.0%). Trauma was the most common etiological factor, observed in 44/75 patients (58.7%). Bursitis or joint effusion (32/75; 42.7%) and rotator cuff tears (29/75; 38.7%) were the most frequently detected pathologies. MRI demonstrated higher sensitivity than USG for detecting overall rotator cuff tears (96.5% vs. 87.0% sensitivity) and full-thickness rotator cuff tears (100% vs. 88.0% sensitivity). MRI also showed higher specificity for rotator cuff tear detection (90.0% vs. 82.0%). Additionally, MRI was superior in detecting labral and instability-related lesions. However, the difference in diagnostic performance between USG and MRI was not statistically significant (χ²=17.07, p=0.105).

Conclusion

USG is a reliable first-line imaging modality for evaluating common shoulder pathologies, while MRI provides superior characterization of complex soft-tissue and intra-articular lesions. A combined imaging approach optimizes diagnostic accuracy and clinical decision-making.

## Introduction

Shoulder pain is one of the most common musculoskeletal complaints encountered in clinical practice and represents a significant cause of functional limitation and reduced quality of life [1]. It is estimated to account for approximately 7-26% of musculoskeletal consultations in the general population, with prevalence increasing with age and occupational overuse [2]. The shoulder joint's wide range of motion (ROM), combined with its complex anatomy and relative instability, predisposes it to a wide spectrum of pathological conditions involving the rotator cuff, biceps tendon, subacromial-subdeltoid (SASD) bursa, labrum, joint capsule, and periarticular soft tissues [3].

The clinical evaluation of shoulder pain is often challenging due to overlapping symptoms among different pathologies [4]. Conditions such as rotator cuff tendinopathy or tears, adhesive capsulitis, subacromial impingement syndrome, calcific tendinitis, bursitis, labral injuries, and degenerative joint disease may present with similar pain patterns and movement restriction [4]. Physical examination tests, although useful, are operator-dependent and may lack sufficient sensitivity and specificity to accurately localize the underlying pathology, particularly in early or subtle disease [5]. Consequently, imaging plays a crucial role in establishing an accurate diagnosis, guiding management, and preventing chronic disability [6].

Ultrasonography (USG) has emerged as a valuable first-line imaging modality in the evaluation of shoulder disorders. It is widely available, cost-effective, noninvasive, and allows dynamic real-time assessment of soft tissue structures [7]. High-resolution musculoskeletal USG enables detailed visualization of the rotator cuff tendons, biceps tendon, bursae, and periarticular soft tissues, making it particularly useful for detecting rotator cuff tears, tendinopathy, bursitis, and calcific deposits [8]. Additionally, the ability to perform dynamic maneuvers during examination enhances its diagnostic utility in impingement syndromes. However, USG is highly operator-dependent and has limited ability to assess deep intra-articular structures, bone marrow abnormalities, and complex labral lesions [9].

Magnetic resonance imaging (MRI) is considered the reference standard for comprehensive evaluation of shoulder pathology. MRI provides excellent soft tissue contrast and multiplanar imaging capability, allowing detailed assessment of both intra-articular and extra-articular structures, including the rotator cuff, labrum, articular cartilage, bone marrow, ligaments, and joint capsule [10]. It is particularly advantageous in diagnosing partial-thickness rotator cuff tears, labral injuries, adhesive capsulitis, occult fractures, and marrow edema. Despite its high diagnostic accuracy, MRI is relatively expensive, time-consuming, and less accessible in resource-limited settings, which may delay diagnosis and treatment [11].

Given the complementary strengths and limitations of USG and MRI, an integrated imaging approach may improve diagnostic accuracy and optimize patient management. In many clinical scenarios, USG serves as an effective screening tool, while MRI provides definitive characterization of complex or equivocal findings [9,11]. However, the choice of imaging modality often depends on clinical suspicion, availability, cost considerations, and expertise, leading to variability in diagnostic pathways across healthcare settings.

There remains a need for prospective observational studies that systematically evaluate the spectrum of shoulder pathologies detected on USG and MRI, assess their relative roles in diagnosing various causes of shoulder pain, and determine how these modalities complement each other in routine clinical practice. Such evidence is particularly relevant in developing healthcare systems, where cost-effective and accurate diagnostic strategies are essential [12]. Therefore, the aim of the present study was to compare the diagnostic performance of USG and MRI in evaluating shoulder pathologies, particularly rotator cuff tears, using surgical or arthroscopic findings as the reference standard where available.

## Materials and methods

Study design and study population

**Figure 1:**
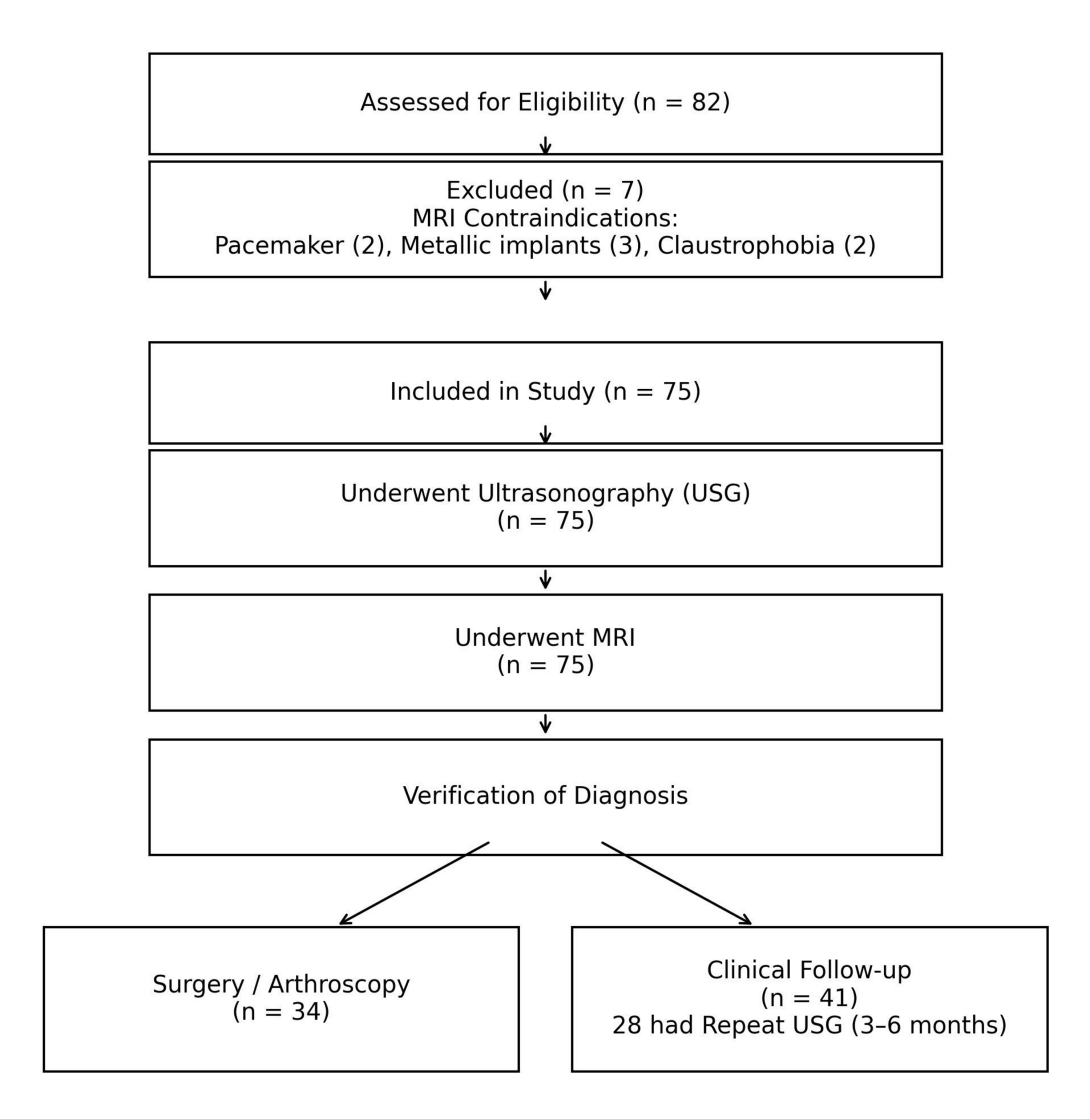
CONSORT flow diagram of patient enrollment, imaging evaluation, and verification pathways CONSORT, Consolidated Standards of Reporting Trials; MRI, magnetic resonance imaging

This prospective observational study was conducted in the Department of Radiodiagnosis at a tertiary care teaching hospital in Central India over a period of approximately two years between June 2023 and June 2025 after obtaining ethical approval from the Institutional Ethics Committee, Government Medical College, Akola (GMC/2023/IEC/008; Date: 07/02/2023). A total of 75 consecutive patients presenting with shoulder-related complaints were included in the study, and all underwent both USG and MRI of the affected shoulder to enable uniform comparison between modalities. Of these, 34 patients underwent surgical intervention or arthroscopy, and intraoperative findings were used as the reference standard for diagnostic correlation. The remaining 41 patients were managed conservatively and followed clinically at three-month intervals for up to one year. Clinical follow-up included assessment of pain severity using the visual analogue scale (VAS), evaluation of ROM in abduction and external rotation, and documentation of functional improvement. Among conservatively treated patients, 28 underwent repeat USG at three to six months based on predefined criteria, including persistent pain (VAS ≥4), restricted ROM, or suspected progression of pathology. Imaging findings were correlated with clinical outcomes to assess concordance. Thus, surgical findings served as the reference standard in operative cases, while structured clinical follow-up with repeat imaging, where indicated, served as the reference standard in nonoperative cases (Figure 1).

Sample size calculation

The sample size was calculated based on the expected sensitivity of USG for detecting rotator cuff tears. The anticipated sensitivity (p) was assumed to be 85% based on previously published literature [7,8]. For diagnostic accuracy analysis, surgical/arthroscopic findings were considered the primary reference standard wherever available. In patients managed conservatively, structured clinical follow-up served as the reference standard. MRI was not used as part of the reference standard in analyses comparing USG and MRI to avoid incorporation bias. Sample size was calculated using the formula: n=Z^2^×p×(1−p)/d^2^; where Z=1.96 (95% CI) and d=0.08 (absolute precision). Substituting the values yielded a minimum required sample size of 68 patients. To account for feasibility and potential exclusions, 75 patients were included in the study.

Inclusion and exclusion criteria

Patients of either sex presenting with shoulder pain, restriction of ROM, or shoulder instability were considered for enrollment. A total of 82 patients were screened during the study period, of whom seven were excluded due to contraindications to MRI, including cardiac pacemakers (n=2), metallic implants (n=3), and severe claustrophobia (n=2). Patients were also excluded if they had isolated bony fractures or bone tumors involving the shoulder region. Individuals with contraindications to MRI, such as cardiac pacemakers, cochlear implants, neurostimulators, aneurysmal clips, infusion pumps, metallic foreign bodies, or severe claustrophobia, were not enrolled. The final study population comprised 75 patients who fulfilled eligibility criteria and underwent both USG and MRI.

Ultrasonography equipment and technique

Ultrasonographic evaluation of the shoulder was performed using a GE LOGIQ P9 ultrasound system (GE Healthcare, Milwaukee, WI, USA) equipped with a high-resolution linear array transducer (frequency range 7.5-15 MHz). Examinations were performed in accordance with standardized musculoskeletal shoulder scanning protocols based on the European Society of Musculoskeletal Radiology (ESSR) and American Institute of Ultrasound in Medicine (AIUM) guidelines [13]. All examinations were conducted by two radiologists with more than seven years of musculoskeletal imaging experience, each having interpreted over 500 shoulder ultrasound examinations prior to the study. Both radiologists had formal training in musculoskeletal imaging during postgraduate and continuing medical education programs.

Patients were examined in a seated position without arm support. The examination protocol included systematic evaluation of the long head of the biceps tendon, subscapularis, supraspinatus, infraspinatus, and teres minor tendons; the SASD bursa; the acromioclavicular joint; greater tuberosity; and the glenohumeral joint. Each structure was assessed in at least two orthogonal planes (longitudinal and transverse). The contralateral asymptomatic shoulder was scanned for comparison. Dynamic maneuvers were performed, including active abduction and internal/external rotation, to assess tendon motion and subacromial impingement. The dynamic impingement sign was operationalized as a binary variable (present/absent) based on failure of normal humeral head depression and visualization of supraspinatus tendon encroachment beneath the acromion during active abduction, as described in prior musculoskeletal ultrasound literature [13].

Radiologists performing USG were blinded to MRI findings at the time of ultrasound examination. MRI interpretations were performed independently and were blinded to ultrasound findings. Clinical history relevant to the symptomatic side was available to both readers, consistent with routine diagnostic practice. Interobserver reliability was not formally assessed in this study.

Ultrasonographic diagnostic criteria

Full-thickness rotator cuff tears were diagnosed when there was complete discontinuity of tendon fibers extending from the articular to the bursal surface, visualized in two orthogonal planes, with or without tendon retraction. Additional imaging signs included a hypoechoic or anechoic defect traversing the entire tendon thickness, direct communication between the glenohumeral joint and the SASD bursa, and visualization of the deltoid muscle or bursal tissue within the defect. These criteria were based on standardized musculoskeletal USG descriptions as recommended by ESSR/AIUM guidelines and prior validation studies [13].

Partial-thickness tears were defined as focal hypoechoic or mixed echogenic defects involving either the articular or bursal surface without complete disruption of the tendon thickness, confirmed in two orthogonal planes. Tear dimensions were measured in longitudinal and transverse planes wherever feasible. The distinction between full- and partial-thickness tears was made solely on imaging criteria. SASD bursitis was diagnosed based on imaging findings of bursal distension with fluid collection and/or bursal thickening greater than 2 mm on static imaging. Clinical tenderness was not used as a diagnostic criterion for bursitis. Biceps tendon pathology was identified based on abnormal echogenicity, tendon thickening, sheath effusion, subluxation, or non-visualization consistent with rupture. All imaging diagnoses were made independently of clinical tenderness assessment to maintain objectivity.

Magnetic resonance imaging protocol

MRI of the shoulder was performed using a 1.5 Tesla scanner (Siemens Magnetom Aera, Siemens Healthineers, Erlangen, Germany) with a dedicated shoulder coil. Patients were positioned supine with the arm in mild external rotation to optimize rotator cuff visualization. Imaging was performed in axial, coronal oblique, and sagittal oblique planes. The standardized imaging protocol included the following sequences (Table 1). The field of view was adjusted to include the acromion superiorly and the surgical neck of the humerus inferiorly. The total scan duration was approximately 20-25 minutes. MRI images were interpreted independently by radiologists who were blinded to USG findings. Clinical history regarding the symptomatic side was available at the time of interpretation, consistent with routine clinical practice. MR (magnetic resonance) arthrography was not routinely performed. In cases of suspected labral pathology, conventional MRI sequences were used for evaluation. MR arthrography was not included due to its invasive nature, additional procedural requirements, cost considerations, and the observational design of the study.

**Table 1:** MRI protocol for shoulder evaluation at 1.5 Tesla TR, repetition time; TE, echo time; PD, proton density; MRI, magnetic resonance imaging

Plane	Sequence	TR (ms)	TE (ms)	Slice thickness	Fat suppression type
Axial	T1-weighted spin echo	500-700	10-20	3-4 mm	None
Axial	PD fat-saturated	2500-3500	30-40	3-4 mm	Spectral fat saturation
Coronal oblique	PD fat-saturated	2500-3500	30-40	3-4 mm	Spectral fat saturation
Coronal oblique	T2-weighted fat-saturated	3000-4000	70-90	3-4 mm	Spectral fat saturation
Sagittal oblique	T1-weighted spin echo	500-700	10-20	3-4 mm	None
Sagittal oblique	PD fat-saturated	2500-3500	30-40	3-4 mm	Spectral fat saturation

Magnetic resonance imaging diagnostic criteria

Rotator cuff tears on MRI were identified by discontinuity of tendon fibers, abnormal signal intensity extending to the articular or bursal surface on proton density (PD) fat-saturated and T2-weighted sequences, tendon retraction, muscle atrophy, and fatty infiltration, evaluated primarily on coronal oblique and sagittal oblique planes. Partial-thickness tears were characterized by focal increased signal intensity not traversing the entire tendon thickness, confirmed in at least two orthogonal planes. Adhesive capsulitis was diagnosed based on a combination of established MRI findings, including capsular and synovial thickening greater than 4 mm at the axillary recess, measured on coronal oblique T2-weighted fat-saturated images, thickening of the coracohumeral ligament (CHL) on sagittal oblique images, and obliteration or infiltration of the rotator interval fat. Additional supportive findings included reduced capsular volume and pericapsular edema on fat-suppressed sequences. These criteria were applied in accordance with previously described MRI-based diagnostic features of adhesive capsulitis [14,15].

Hill-Sachs lesions were identified as wedge-shaped cortical defects involving the posterolateral humeral head, best visualized on axial and coronal oblique sequences, with or without associated marrow edema on fat-suppressed images. Labral pathologies such as superior labrum anterior to posterior (SLAP) and Bankart lesions were diagnosed based on abnormal labral morphology, detachment, high-signal clefts extending to the labral surface on PD/T2 fat-saturated images, or associated bony defects [16]. Calcific tendinitis was identified as a focal hypointense signal on all sequences with surrounding inflammatory changes. All measurements were performed on the sequences and planes specified above to ensure consistency and reproducibility.

SASD bursitis on USG was defined as bursal distension with fluid collection and/or bursal thickening exceeding 2 mm on static imaging. On MRI, bursitis was defined as fluid distension of the SASD bursa with a hyperintense signal on T2-weighted or fat-saturated sequences. Glenohumeral joint effusion on USG was defined as anechoic or hypoechoic fluid distending the joint recess, particularly in the posterior or axillary recess. On MRI, joint effusion was identified as hyperintense intra-articular fluid on T2-weighted or fat-suppressed sequences exceeding physiologic volume.

Correlation and follow-up

Ultrasonographic findings were compared with MRI findings in all 75 patients. Of these, 34 patients underwent surgical intervention or arthroscopy, and imaging findings from both USG and MRI were correlated with intraoperative observations, which served as the reference standard in these cases. The remaining 41 patients were managed conservatively and followed clinically for up to one year. Among these, 28 patients underwent repeat USG at three to six months based on predefined criteria, including persistent pain (VAS ≥4) or restricted ROM. In nonoperative cases, imaging findings were correlated with clinical progression or improvement during follow-up.

Statistical analysis

Data were entered into Microsoft Excel and analyzed using the Statistical Package for the Social Sciences (SPSS) version 20.0 (IBM Corp., Armonk, NY). Descriptive statistics were used to summarize demographic characteristics, clinical presentation, and imaging findings, with categorical variables expressed as frequencies and percentages. Diagnostic accuracy measures, including sensitivity and specificity, were calculated exclusively in the surgically verified subgroup (n=34), using operative findings as the reference standard. For each pathology, sensitivity was calculated using the number of surgically confirmed positive cases as the denominator, and specificity was calculated using the number of surgically confirmed negative cases within this subgroup. Ninety-five percent CI were calculated using the Wilson method for proportions. Agreement between USG and MRI was assessed descriptively. The chi-square (χ²) test was applied for comparisons of categorical variables where appropriate. A p-value of less than 0.05 was considered statistically significant.

## Results

The study population comprised 75 patients, with a male predominance (69.3%) compared to females (30.7%). The most common age group affected was 20-29 years (32.0%), followed by patients aged ≥50 years (28.0%) and 40-49 years (22.7%). Shoulder involvement was more frequently observed on the right side (72.0%) than on the left (28.0%), indicating a predominance of dominant-side involvement in the study cohort (Table 2).

**Table 2:** Baseline demographic characteristics of the study population (n=75)

Variable	Frequency	%
Gender		
Male	52	69.3
Female	23	30.7
Age group (years)		
10-19	4	5.3
20-29	24	32.0
30-39	9	12.0
40-49	17	22.7
≥50	21	28.0
Side involved		
Right	54	72.0
Left	21	28.0

Shoulder pain was the most common presenting symptom, either alone (32.0%) or in combination with restricted ROM (28.0%). Restriction of abduction alone was observed in 25.3% of patients, while shoulder dislocation constituted 14.7% of presentations. Only 10.7% of patients were sportspersons, whereas the majority (89.3%) were non-sportspersons. A traumatic etiology was identified in 58.7% of cases, while 41.3% had nontraumatic causes of shoulder pathology (Table 3).

**Table 3:** Clinical presentation, sports activity, and etiopathology of shoulder disorders ROM, range of motion

Variable	Frequency	%
Presenting symptoms		
Shoulder pain alone	24	32.0
Restriction of movement (abduction only)	19	25.3
Both pain and restricted ROM	21	28.0
Dislocation	11	14.7
Sports activity		
Sportsperson	8	10.7
Non-sportsperson	67	89.3
Etiopathology		
Traumatic	44	58.7
Non-traumatic	31	41.3

**Figure 2:**
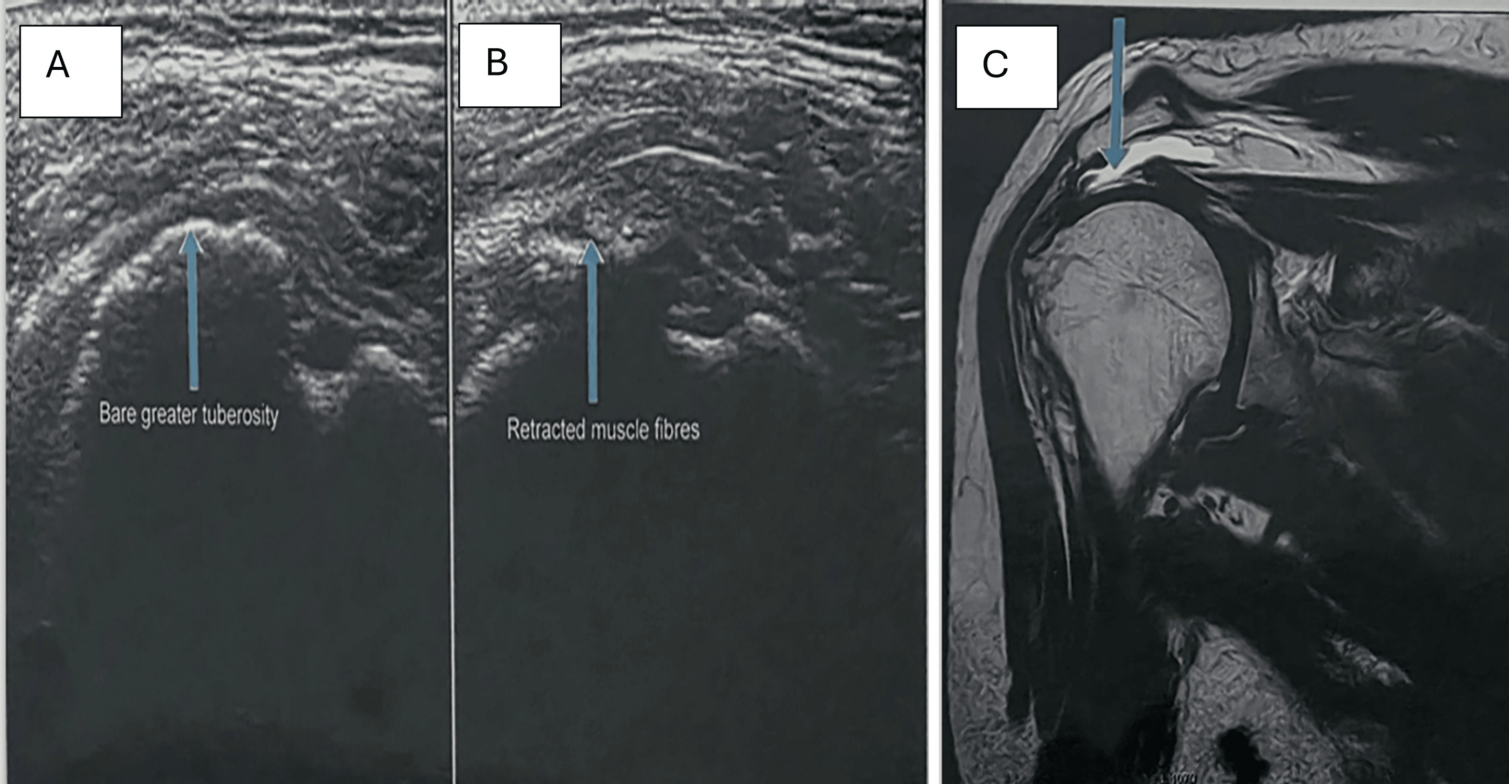
Full-thickness supraspinatus tendon tear on USG and MRI. (A-B) USG showing discontinuity and retraction of supraspinatus tendon fibers with exposure of the greater tuberosity ("bare tuberosity sign"). (C) Coronal T2-weighted MRI image showing nonvisualization of the supraspinatus tendon with proximal tendon retraction (arrow) and mild subacromial bursal effusion USG, ultrasonography; MRI, magnetic resonance imaging

**Figure 3:**
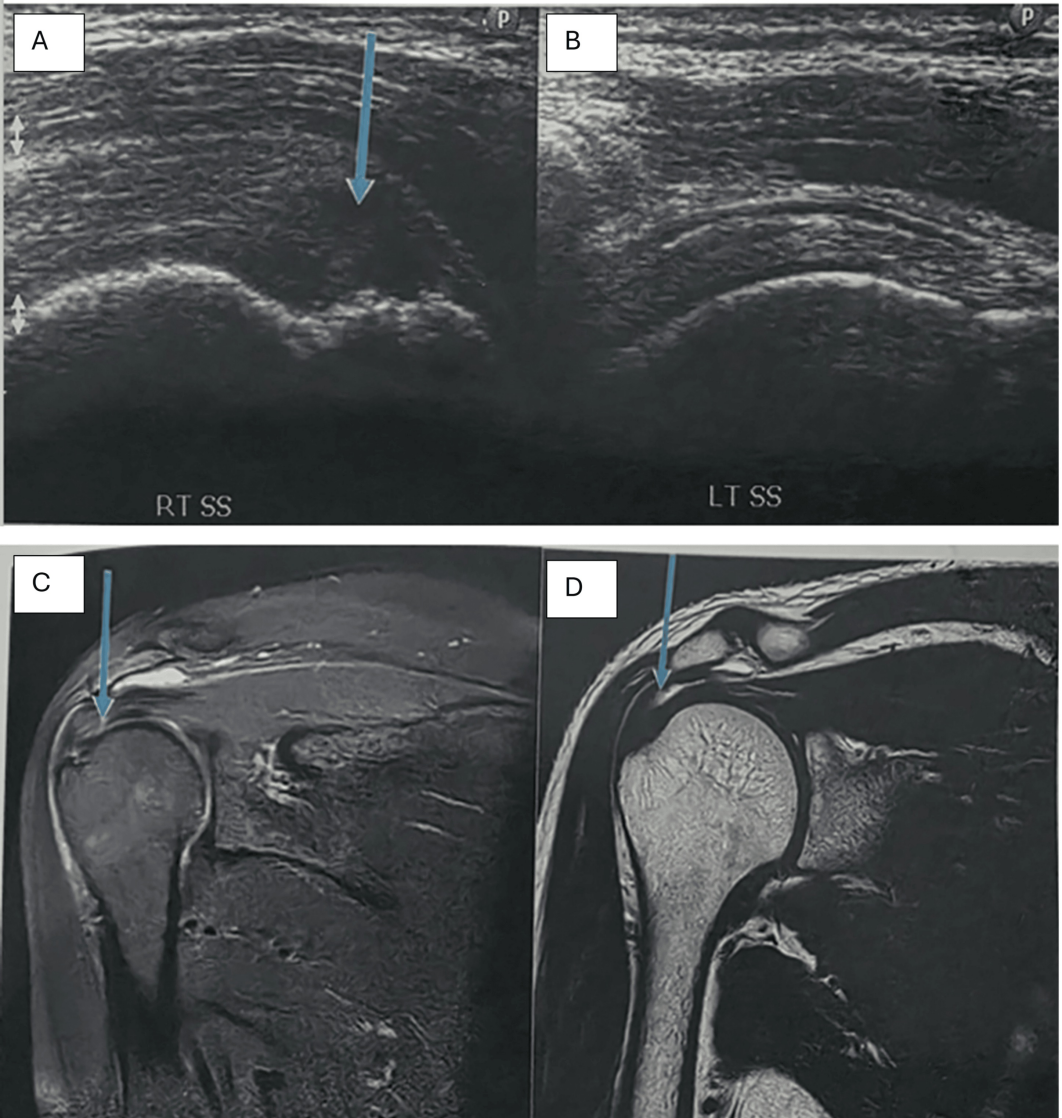
Partial-thickness supraspinatus tendon tear. (A-B) USG showing hypoechoic thickening in the supraspinatus tendon near its insertion over the greater tuberosity (arrow). (C-D) PD-weighted and T2-weighted MRI confirming a partial-thickness tear at the supraspinatus tendon insertion USG, ultrasonography; MRI, magnetic resonance imaging; PD, proton density

**Figure 4:**
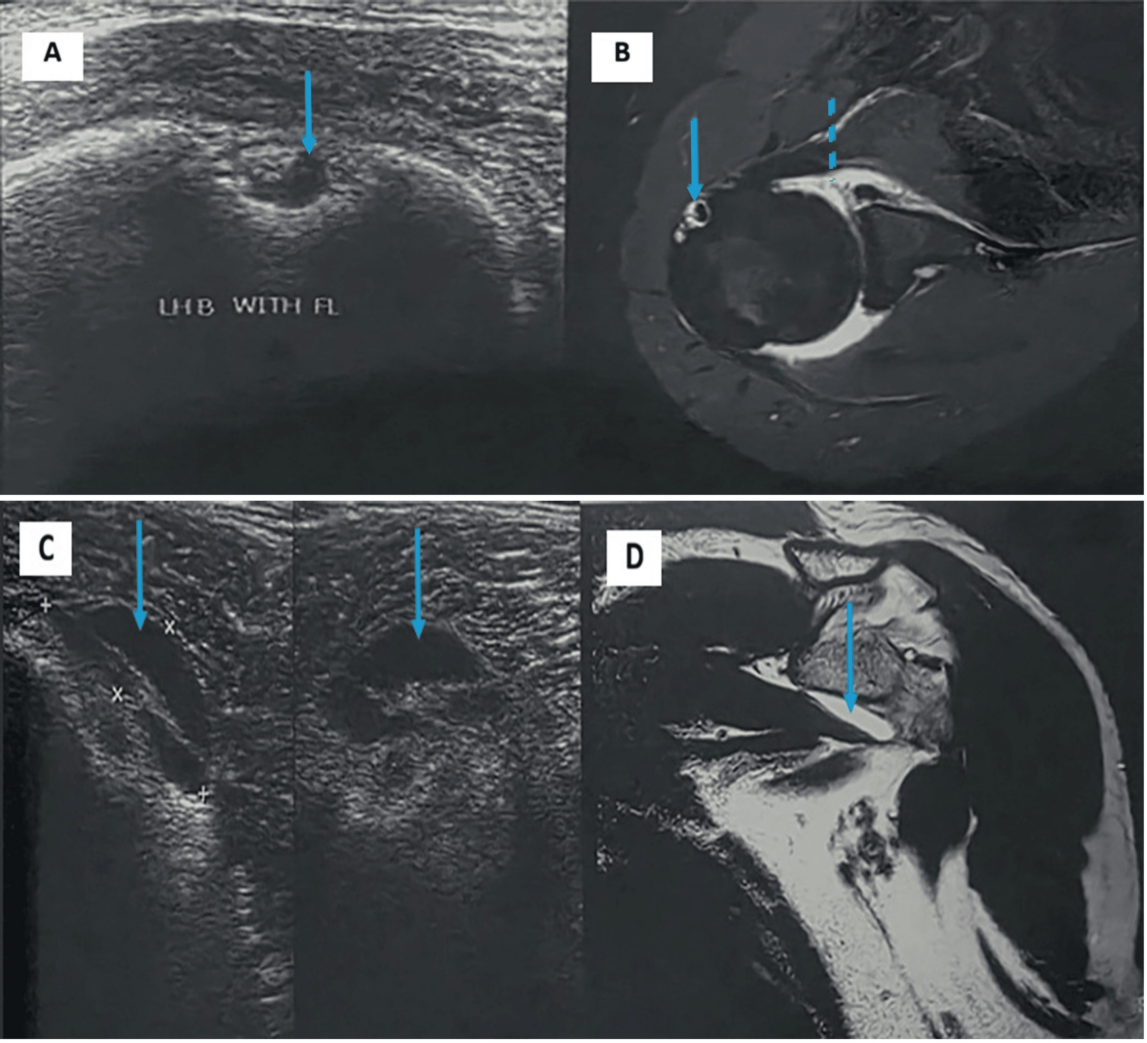
(A-B) Joint effusion with fluid in the sheath of the LHB tendon - biceps tendinitis. (A) Transverse view of the biceps on USG showing fluid in the biceps sheath (solid arrow). (B) Corresponding axial MRI PDFS image showing fluid in the biceps sheath (solid arrow) and mild joint effusion (dotted arrow). (C) Subcoracoid bursitis - USG shows a pocket of fluid in the subcoracoid region measuring 2.2x0.7 cm s/o subcoracoid bursitis. (D) Small T2-weighted hyperintense fluid collection seen in the subcoracoid region on coronal images (bursa=2x0.5 cm) s/o subcoracoid bursitis PDFS, proton density-weighted fat-saturated; LHB, long head of the biceps; USG, ultrasonography; MRI, magnetic resonance imaging; LHB, long head of the biceps

SASD bursitis and glenohumeral joint effusion were analyzed as separate entities. SASD bursitis was identified in 18 patients (24.0%) on USG and 20 patients (26.7%) on MRI, while glenohumeral joint effusion was detected in 12 patients (16.0%) on USG and 12 patients (16.0%) on MRI, with corresponding findings on surgical or follow-up correlation. Partial-thickness rotator cuff tears were detected equally by USG, MRI, and surgery in 20.0% of patients. Full-thickness tears were detected in 13.3% on USG, whereas MRI and surgical findings demonstrated a higher detection rate of 17.3%. Rotator interval tears and supraspinatus muscle atrophy were more accurately identified on MRI and surgery than on USG (Table 4 and Figures 2-4).

**Table 4:** Comparison of rotator cuff-related and associated findings on USG, MRI, and surgical/follow-up correlation USG, ultrasonography; MRI, magnetic resonance imaging; SASD, subacromial-subdeltoid

Pathology	USG	MRI	Surgery/arthroscopy/ Follow-up
	Frequency (%)		
Full-thickness tear	10 (13.3)	13 (17.3)	13 (17.3)
Partial-thickness tear	15 (20.0)	15 (20.0)	15 (20.0)
Rotator interval tear	1 (1.3)	3 (4.0)	3 (4.0)
SASD bursitis	18 (24.0)	20 (26.7)	20 (26.7)
Glenohumeral joint effusion	12 (16.0)	12 (16.0)	12 (16.0)
Supraspinatus muscle atrophy	6 (8.0)	7 (9.3)	7 (9.3)

**Figure 5:**
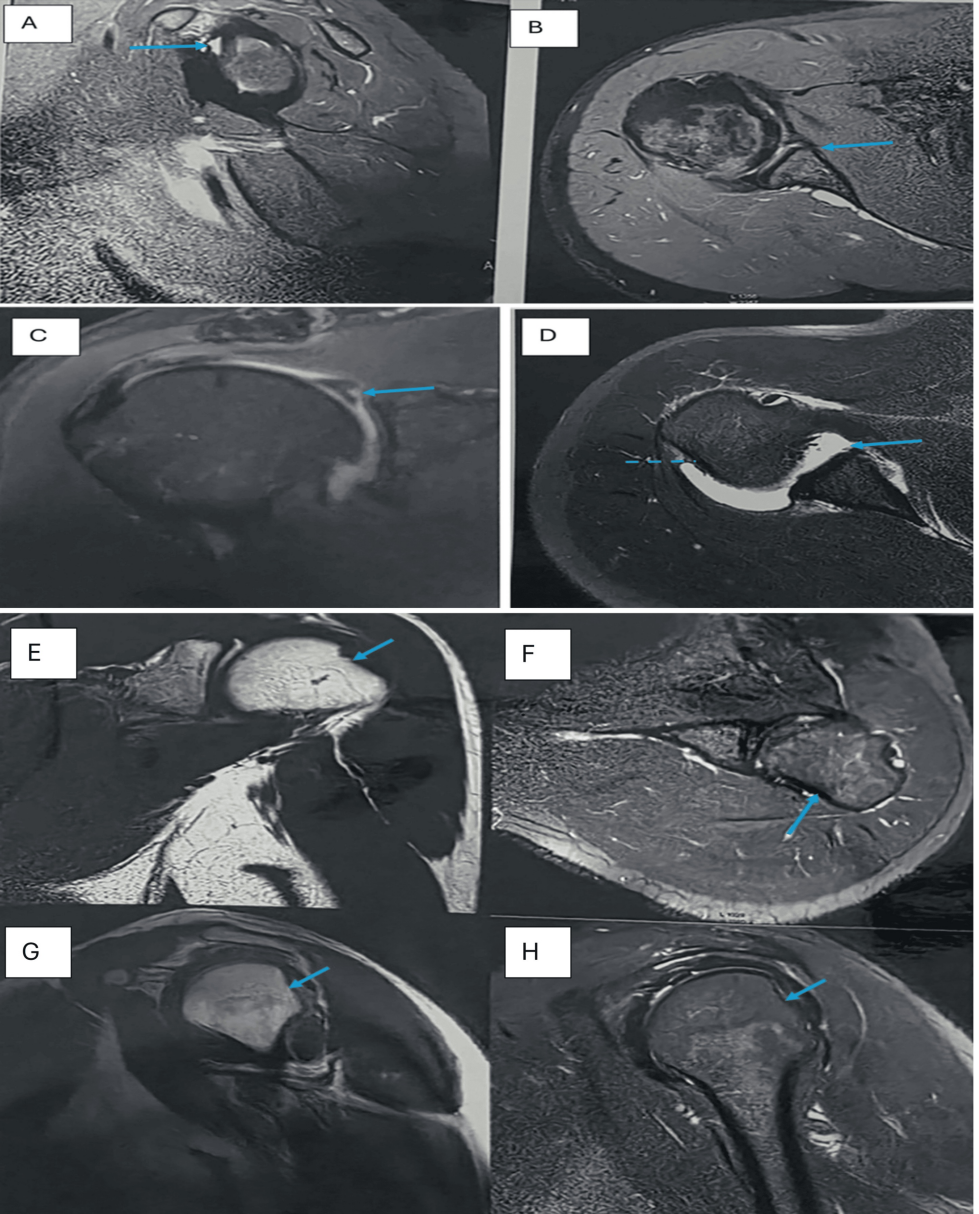
Labral and instability-related lesions on MRI PDFS images: (A-B) ALPSA lesion showing linear hyperintensity along the periosteum of the anterior glenoid. The labrum appears intact. (C) Type 2 SLAP lesion showing linear hyperintensity in the superior labrum. (D) Bankart lesion with attenuation and hyperintensity in the anteroinferior glenoid labrum (solid arrow) and associated cortical flattening of the posterosuperior aspect of the humeral head (dotted arrow), suggesting Hill-Sachs deformity ALPSA, anterior labroligamentous periosteal sleeve avulsion; SLAP, superior labrum anterior to posterior; PDFS, proton density-weighted fat-saturated; MRI, magnetic resonance imaging

**Figure 6:**
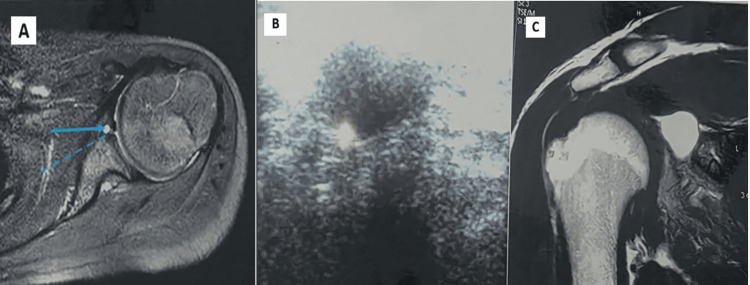
Labral cyst: Axial PDFS MRI image showing a tiny cystic lesion (solid arrow) along the anterior labrum with linear hyperintensity (dotted arrow) in the anteroinferior labrum, suggesting a labral tear. Paralabral cyst (B-C): (B) USG showing a well-defined, rounded cystic lesion in the paralabral region beneath the supraspinatus toward the suprascapular notch. (C) T2-weighted coronal MRI image demonstrating a paralabral cyst (arrow) arising from a labral tear (not shown) USG, ultrasonography; MRI, magnetic resonance imaging; PDFS, proton density-weighted fat-saturated

**Figure 7:**
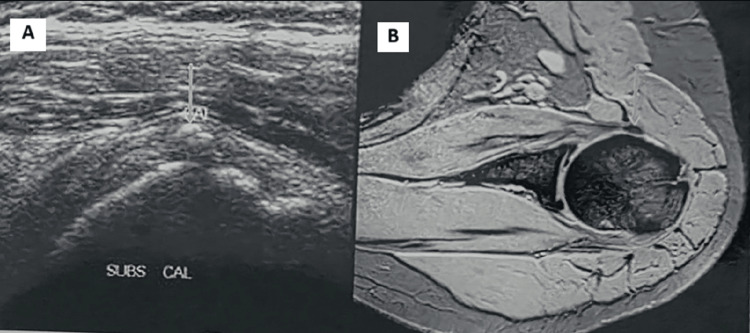
Calcific tendinitis involving the subscapularis tendon: (A) USG showing calcific density foci in the subscapularis tendon near its insertion. (B) Corresponding axial T2FFE MRI image showing blooming in the subscapularis tendon near its insertion T2FFE, T2-weighted fast field echo; USG, ultrasonography; MRI, magnetic resonance imaging

**Figure 8:**
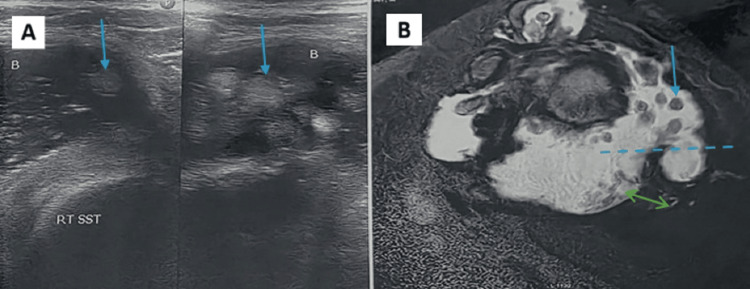
Synovial chondromatosis: (A) USG showing gross joint effusion with multiple echogenic loose bodies (arrow). (B) Coronal PDFS MRI image showing gross joint effusion (dotted arrow) with synovial thickening (double-headed arrow) and multiple hypointense loose bodies (solid arrow) USG, ultrasonography; MRI, magnetic resonance imaging; PDFS, proton density-weighted fat-saturated

MRI demonstrated superior detection of instability-related lesions, including Hill-Sachs lesions (10.7%) (Figure 5D-5H), Bankart lesion (9.3%) (Figure 5D), anterior labroligamentous periosteal sleeve avulsion (ALPSA) lesion (2.7%) (Figure 5A-5B), and SLAP lesion (1.3%) (Figure 5C), many of which were not identified on USG (Table 5 and Figure 5). Tiny labral cysts were not identified on USG due to being deep and small; however, paralabral cysts were seen on USG (Figure 6B). MRI demonstrated both labral and paralabral cysts (Figure 6). Calcific tendinitis was seen equally on USG and MRI (Figure 7). Miscellaneous or rare pathologies such as synovial osteochondromatosis, quadrilateral space syndrome, and adhesive capsulitis were identified with nearly equal accuracy across MRI and USG (Figure 8).

**Table 5:** Distribution of shoulder pathologies detected by USG, MRI, and surgery The p-value represents the comparison of detection rates of shoulder pathologies between USG and MRI using the chi-square test (χ²=17.07, df=11, p=0.105). Surgical/arthroscopic and follow-up findings were considered the reference standard. A p-value of <0.05 was considered statistically significant. USG, ultrasonography; MRI, magnetic resonance imaging; AC, acromioclavicular; ALPSA, anterior labroligamentous periosteal sleeve avulsion; SLAP, superior labrum anterior to posterior

Pathology	USG	MRI	Surgery/arthroscopy/follow-up
	Frequency (%)		
Rotator cuff tear (overall)	25 (33.3)	28 (37.3)	29 (38.7)
Acromioclavicular arthrosis	16 (21.3)	18 (24.0)	18 (24.0)
Hill-Sachs lesion	3 (4.0)	8 (10.7)	8 (10.7)
Bankart lesion	0 (0.0)	7 (9.3)	7 (9.3)
Labral cyst	2 (2.7)	3 (4.0)	3 (4.0)
Calcific tendinitis	3 (4.0)	2 (2.7)	3 (4.0)
Muscle edema	2 (2.7)	2 (2.7)	2 (2.7)
ALPSA lesion	0 (0.0)	2 (2.7)	2 (2.7)
Synovial osteochondromatosis	1 (1.3)	1 (1.3)	1 (1.3)
Adhesive capsulitis	0 (0.0)	1 (1.3)	1 (1.3)
SLAP lesion	0 (0.0)	1 (1.3)	1 (1.3)
Quadrilateral space syndrome	1 (1.3)	1 (1.3)	1 (1.3)

MRI demonstrated higher diagnostic performance than USG in the surgically verified subgroup (n=34), where operative findings served as the reference standard. For overall rotator cuff tears, MRI showed a sensitivity of 96.5% and specificity of 90.0%, compared with 87.0% sensitivity and 82.0% specificity for USG. For full-thickness tears, MRI achieved 100% sensitivity and specificity, whereas USG demonstrated 88.0% sensitivity and 81.0% specificity. In partial-thickness tears, MRI sensitivity (94.0%) exceeded that of USG (86.0%), with comparable specificity between the two modalities. Although MRI reached 100% sensitivity and specificity for full-thickness tears, the corresponding confidence intervals were wide due to the limited sample size (Table 6).

**Table 6:** Diagnostic performance of USG and MRI for rotator cuff pathologies in the surgically verified subgroup (n=34) Sensitivity and specificity were calculated using surgical/arthroscopic findings as the reference standard in the surgically verified subgroup (n=34). Ninety-five percent CI were calculated using the Wilson method. TP, true positive; FN, false negative; TN, true negative; FP, false positive; USG, ultrasonography

Pathology	Modality	Sensitivity % (95% CI)	Specificity % (95% CI)
Rotator cuff tear (overall)	MRI	96.5% (82.2-99.9)	90.0% (55.5-99.7)
	USG	87.0% (69.3-96.2)	80.0% (37.6-96.4)
Full-thickness tear	MRI	100% (75.3-100)	100% (83.9-100)
	USG	84.6% (54.6-98.1)	81.0% (58.1-94.6)
Partial-thickness tear	MRI	93.3% (68.1-99.8)	84.2% (60.4-96.6)
	USG	86.7% (59.5-98.3)	84.2% (60.4-96.6)

## Discussion

In this prospective observational study, USG and MRI were systematically evaluated for diagnosing shoulder pathologies, with surgical or arthroscopic correlation where available. The study population demonstrated a clear male predominance (69.3%) and higher right-sided involvement (72.0%), findings that are consistent with previous Indian and international studies by Nunna et al. and Zheng et al. and likely reflect greater involvement of the dominant shoulder in occupational and daily activities [17,18]. The predominance of younger and middle-aged adults, particularly those in the 20-29 and ≥50-year age groups, suggests a dual burden of traumatic injuries in younger individuals and degenerative pathology in older patients, as described in an earlier epidemiological report by Zoga et al. [19].

Trauma was identified as the leading etiological factor (58.7%), and shoulder pain, either alone or associated with restricted ROM, was the most frequent presenting symptom. This clinical profile aligns with prior studies by Zoga et al. and Barad et al. that report trauma and overuse as major contributors to rotator cuff and instability-related shoulder disorders [19,20]. The relatively lower proportion of sportspersons (10.7%) highlights that shoulder pathology in the Indian setting is more commonly related to occupational strain and accidental trauma rather than organized sports injuries [21].

Analysis of imaging findings revealed that bursitis or joint effusion was the most common abnormality detected across all modalities, followed by rotator cuff tears. USG demonstrated good detection of superficial soft-tissue abnormalities such as bursitis, tendinopathy, and partial-thickness rotator cuff tears. However, MRI consistently identified a higher number of full-thickness tears, rotator interval tears, and supraspinatus muscle atrophy, findings that were corroborated by surgical observations. This difference can be explained by MRI’s superior soft-tissue contrast and multiplanar capability, which allows better visualization of deep and complex anatomical structures, tendon retraction, and muscle degeneration [22,23].

MRI demonstrated clear superiority in detecting labral and instability-related lesions, including Bankart (9.3%), ALPSA (2.7%), and SLAP (1.3%) lesions, many of which were not identified on USG. This finding is expected, as USG has inherent limitations in evaluating deep intra-articular structures such as the glenoid labrum due to acoustic shadowing and limited visualization of the glenohumeral joint recesses. Therefore, absence of labral pathology on USG should not be interpreted as true absence clinically, particularly in patients with instability or persistent symptoms. Similar observations have been reported by Chauhan et al. and Madhavi et al., who emphasized the limited role of USG in evaluating labral and capsulolabral lesions due to acoustic shadowing and restricted visualization of the glenoid labrum [24,25]. The identification of rare entities such as synovial osteochondromatosis and quadrilateral space syndrome further supports MRI’s role as a comprehensive diagnostic tool in complex shoulder pathology.

In terms of diagnostic performance, MRI showed higher sensitivity and specificity than USG for overall rotator cuff tears (96.5% vs. 87% sensitivity) and achieved 100% sensitivity and specificity for full-thickness tears. These findings are comparable to those reported by Ganesh et al., who demonstrated MRI sensitivity approaching 95-100% for full-thickness rotator cuff tears, while USG showed slightly lower sensitivity but remained clinically useful [26]. For partial-thickness tears, both modalities showed reduced sensitivity compared to full-thickness tears, reflecting the inherent diagnostic difficulty of these lesions due to subtle tendon fiber disruption.

Despite MRI demonstrating numerically superior diagnostic accuracy, the Chi-square analysis revealed no statistically significant difference between MRI and USG (p=0.460). This lack of statistical significance may be attributed to the relatively small sample size and overlapping diagnostic capabilities of the two modalities for common pathologies. Similar observations have been reported in previous comparative studies by Pandya et al. and Rathi et al., which concluded that while MRI is diagnostically superior, high-resolution USG performed by experienced operators provides comparable results for many rotator cuff disorders [27,28].

Based on the present findings, a practical imaging approach may be recommended: USG may be used as a first-line modality for evaluation of rotator cuff pathology, bursitis, and biceps tendon abnormalities due to its accessibility and dynamic capability. However, in cases of suspected instability, recurrent dislocation, persistent symptoms despite negative USG, or clinical suspicion of labral pathology, MRI should be performed for comprehensive intra-articular assessment.

Taken together, the findings of this study support the use of USG as an effective first-line imaging modality for evaluating shoulder pain, particularly in resource-limited settings, owing to its accessibility, cost-effectiveness, and dynamic assessment capability. MRI, however, remains indispensable for comprehensive evaluation, especially in cases of suspected full-thickness tears, muscle atrophy, labral lesions, and instability-related pathologies. An integrated imaging approach, utilizing USG for initial assessment and MRI for problem-solving and preoperative planning, appears to be the most rational diagnostic strategy [7,29].

Limitations

This study has several limitations. First, the sample size was modest, which may have limited statistical power despite observed differences in diagnostic performance. Second, although standardized imaging protocols were followed, USG is inherently operator-dependent, and interobserver variability was not formally assessed, which may affect the reproducibility of diagnostic accuracy estimates. Third, surgical or arthroscopic confirmation was available only in a subset of patients, introducing potential verification bias. Fourth, although USG and MRI were interpreted independently, radiologists were not blinded to relevant clinical history, which may have introduced interpretation bias. Fifth, the study cohort included a relatively high proportion of young and trauma-related cases, raising the possibility of spectrum bias that may influence diagnostic performance estimates when extrapolated to older or predominantly degenerative populations. Sixth, patients with contraindications to MRI were excluded prior to enrollment. As all participants were required to undergo both USG and MRI, the study population was inherently limited to MRI-eligible individuals. Although only seven patients were excluded due to MRI contraindications, this may have influenced generalizability to patients with implanted devices or severe claustrophobia. Seventh, advanced MRI techniques such as MR arthrography were not utilized, which could have improved the detection of subtle labral and capsuloligamentous lesions. Finally, as a single-center study conducted in a tertiary-care setting, the findings may not be fully generalizable to other healthcare environments.

## Conclusions

USG and MRI demonstrate complementary roles in the evaluation of shoulder pathologies. In the present study, MRI showed higher numerical sensitivity and specificity for detecting rotator cuff tears and intra-articular lesions such as labral and instability-related abnormalities, while USG effectively identified common rotator cuff abnormalities and superficial soft-tissue conditions. However, the differences between the two modalities did not reach statistical significance. Considering its accessibility, dynamic capability, and lower cost, USG can serve as an effective first-line imaging modality for the initial assessment of shoulder pain. MRI remains valuable for the comprehensive evaluation of complex, equivocal, or intra-articular pathologies and for preoperative planning.
